# Optimizing the fragment complementation of APEX2 for detection of specific protein-protein interactions in live cells

**DOI:** 10.1038/s41598-017-12365-9

**Published:** 2017-09-27

**Authors:** Miaomiao Xue, Junjie Hou, Linlin Wang, Dongwan Cheng, Jingze Lu, Li Zheng, Tao Xu

**Affiliations:** 1 National Laboratory of Biomacromolecules, CAS Center for Excellence in Biomacromolecules, Institute of Biophysics, Chinese Academy of Sciences, Beijing, 100101 China; 2 College of Life Sciences, University of Chinese Academy of Sciences, Beijing, 100049 China

**Keywords:** Proteins, Proteomics, Enzymes

## Abstract

Dynamic protein-protein interactions (PPIs) play crucial roles in cell physiological processes. The protein-fragment complementation (PFC) assay has been developed as a powerful approach for the detection of PPIs, but its potential for identifying protein interacting regions is not optimized. Recently, an ascorbate peroxidase (APEX2)-based proximity-tagging method combined with mass spectrometry was developed to identify potential protein interactions in live cells. In this study, we tested whether APEX2 could be employed for PFC. By screening split APEX2 pairs attached to FK506-binding protein 12 (FKBP) and the FKBP12-rapamycin binding (FRB) domain, which interact with each other only in the presence of rapamycin, we successfully obtained an optimized pair for visualizing the interaction between FRB and FKBP12 with high specificity and sensitivity in live cells. The robustness of this APEX2 pair was confirmed by its application toward detecting the STIM1 and Orial1 homodimers in HEK-293 cells. With a subsequent mass spectrometry analysis, we obtained five different biotinylated sites that were localized to the known interaction region on STIM1 and were only detected when the homodimer formed. These results suggest that our PFC pair of APEX2 provides a potential tool for detecting PPIs and identifying binding regions with high specificity in live cells.

## Introduction

Protein-protein interactions are dynamic and are precisely regulated to conduct many physiological functions within cells. Dysregulated interactions are related to dysfunction and lead to many diseases, including cancer, diabetes and neurodegenerative disorders^1–4^. Mapping the comprehensive protein interactome and identifying the key interaction regions between binding partners in health and disease are major tasks for life science investigations.

Proximity-dependent biotin labeling by modifying enzymes, such as tyramide^5,6^, ascorbate peroxidase (APEX)^7–9^, and BirA^10^, is a recently developed method for protein-protein interaction screening that addresses this challenge. APEX2, with quadruple mutants of soybean APEX, has an improved enzyme activity for biotinylating proteins within 20 nm and can increase electron microscope (EM) contrasts by catalyzing the polymerization of 3,3′-diaminobenzidine (DAB)^7,9^. When fused to the protein of interest, APEX2, in the initiation of hydrogen peroxide, can catalyze biotin-phenol to produce a biotin-phenol radical that is unstable and can covalently bind to neighboring proteins, which allows their subsequent purification and identification by mass spectrometry (MS).

Protein fragment complementation (PFC) assays have been widely used and shown to be powerful in the detection of protein-protein interactions in intact cells and in live animals^11–13^. All PFCs that are based on easy readouts, such as enzyme activity, transcription activation state or fluorescence, require stable PPIs for reconstitution^11,14,15^. Although they have several advantages in the detection of the interaction in the live state, none of them can directly label the interaction partners. The recently developed split HRP, which can catalyze biotinylation in live cells, has potential for direct labeling but is not suitable for interactions within cells, a consequence of spontaneous reconstitution in the endoplasmic reticulum (ER)^16^.

For the identification of the key regions between a protein and its partners, several approaches were developed including the X-ray crystallography, NMR spectroscopy and cross-linking of proteins combined with mass spectrometry. However, the study of the physiological binding feature still faces multiple challenges, such as the complexity of the sample purification procedures^17^.

In this study, taking the advantages of the APEX2 enzyme, we carried out a screen for an optimized PFC of APEX2 to enhance the specificity of the catalytic activity of the enzyme. Employing a rapamycin-induced interaction pair, FRB and FKBP12, we screened truncated APEX2 mutants based on structure and finally obtained one optimized complementary pair that lost enzymatic activity when separated and regained it only when they were brought closer together. We also validated the specificity of this strategy in two independent experiments, using homodimers of STIM1 and Orai1. Although they both can form self-associated homodimers independently of the activation state, only STIM1 can bring the PFC to reconstitute the APEX2 activity. The failure to reconstitute the activity by the Orai1 homodimer could be attributed to its C-terminal steric constraints. By use of streptavidin-based affinity purification and subsequent tandem MS, we successfully identified 18 biotinylated peptides in STIM1. More importantly, these biotinylation sites were highly enriched within the known interaction region on STIM1. Thus, this strategy is a useful tool for uncovering protein-protein interactions under specific conditions and mapping the interface of the complex in living cells.

## Results

### Screening the PFC of APEX2 through the rapamycin-induced protein interaction

**Figure 1:**
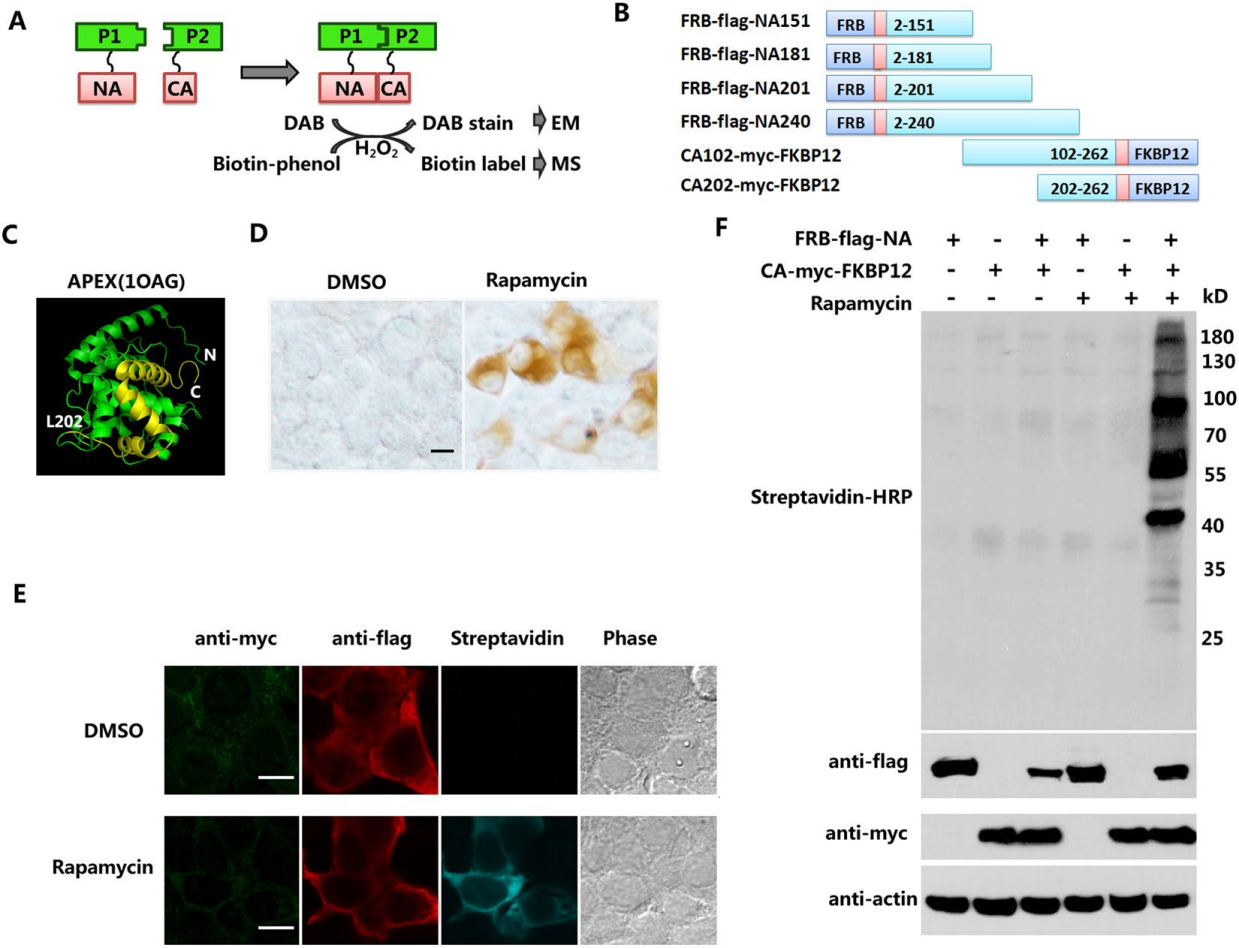
Optimization of the N-terminal APEX2(NA)/C-terminal APEX2(CA) complementation pair in cells. (**A**) Schematic of protein-fragment complementation (PFC) of APEX2. The interaction between proteins 1 (P1) and 2 (P2) brings inactive NA and CA into close proximity to reconstitute the peroxidase activity. This enzyme can biotin-label neighboring proteins within 20 nm for MS and catalyze the polymer of DAB for EM imaging. (**B**) The initial PFCs fused with FRB-flag and myc-FKBP12 to generate incremental truncation libraries. (**C**) Structure of wild-type soybean ascorbate peroxidase (1OAG). The NA and CA fragments are highlighted in green and yellow, respectively. (**D**) DAB staining for the rapamycin-induced FRB/FKBP12 association in live cells. HEK-293 cells were transfected with FRB-flag-NA and CA-myc-FKBP12, followed by treatment with rapamycin or DMSO as indicated. DAB staining was performed as described. Scale bars, 10 μm. (**E**) Biotin labeling was tested using the confocal image. HEK-293 cells were transfected with different plasmids as indicated and treated with rapamycin or DMSO. Biotin labeling was initiated with H_2_O_2_ following biotin-phenol incubation. Scale bars, 10 μm. (**F**) Western blot to test biotin labeling in the live cells. Cells were treated as in (**E**). The whole lysates were resolved by 10% SDS-PAGE and tested by streptavidin-HRP for biotinylated proteins, anti-flag antibody for FRB-NA and anti-myc antibody for CA-FKBP12. Equal amount of protein in each lane was loaded as quantified by β-actin. The original blots were presented in the Supplementary Fig. S3.

The rapamycin-binding domain of the mammalian target of rapamycin (FRB) can bind to FK506-binding protein 12 (FKBP) in the presence of rapamycin with high affinity^18^. Employing this property, we constructed sets of truncations for optimizing the APEX2 complementary pairs which fused to FRB and FKBP12. Basically, the different truncation pairs that fused to the FRB or FKBP12 were spaced by a linker^12^. Because the APEX2 enzyme can catalyze the H_2_O_2_-dependent polymerization of DAB, leading to localized precipitation^19,20^, it is easy to visualize the complementation by common light microscopy (Fig. 1A). The criterion is that DAB staining is exclusively produced when the PFC of APEX2 bind together in the presence of rapamycin, while there is no detectable signal when expressed alone or when both are expressed but without rapamycin.

The crystal structures of APEX from the pea and soybean demonstrate the heme-binding sites and ascorbate-binding sites form the key catalytic region^21–23^. The Cys32 and Arg172 residues are ascorbate-binding sites, and there are 14 helices and 4 beta sheets in the whole APEX2 (1OAG) protein secondary structure^23^. Based on this information, we began with four N-terminal truncations (NA151, NA181, NA201 and NA240), all of which avoided disruptions to the helices, except NA240, together with two C-terminal truncations (CA102 and CA202) that had some overlap with the N-terminal mutants (Fig. 1B). We were lucky to observe that the NA201 and CA202 pairs worked well, while the others were not satisfactory (Supplementary Fig. S1). To determine if there were any other sites suitable for the PFC, we carried out a second screening around NA201 and Arg 172, which played a major role in the stabilization of substrate binding (Supplementary Fig. S1). In the end, we obtained 3 NAs (NA201, NA215, NA230) that could reconstitute the enzyme with CA202 without spontaneous activity. Among these, we selected the NA201/CA202 pair, hereafter referred to as NA/CA, which had the highest enzyme activity with the lowest background.

The optimized NA and CA are indicated in green and yellow in the map of the APEX2 structure (Fig. 1C). There were significant DAB signals in the HEK-293 cells co-expressing the FRB-flag-NA/CA-myc-FKBP12 in the presence of rapamycin, while few detectable signals over the background were evident with the DMSO treatment (Fig. 1D). We then tested the biotinylation ability of this pair. The biotinylated proteins in the HEK-293 cells expressing both FRB-flag-NA and CA-myc-FKBP12 were predominantly detectable with streptavidin-cy5 only in the presence of rapamycin compared to the cells treated with DMSO (Fig. 1E). These results were confirmed by a western blot experiment with a streptavidin-HRP probe (Fig. 1F). The expression levels of FRB-flag-NA/CA-myc-FKBP12 were tested using anti-flag and anti-myc antibodies, respectively (Fig. 1F). These results confirmed that the NA/CA pair had specific enzymatic activity in both DAB staining and biotin labeling. We then applied the pair to two other independent cases of protein-protein interaction to examine the specificity of our complementary fragments of APEX2 for the detection of dimerization.

### Detection of homodimer interaction

To lend support to the specificity of the APEX2 pair, we checked its performance in two cases: homodimers of stromal interacting molecule 1 (STIM1) and Orai1, respectively.

### STIM1 Homodimerization

**Figure 2:**
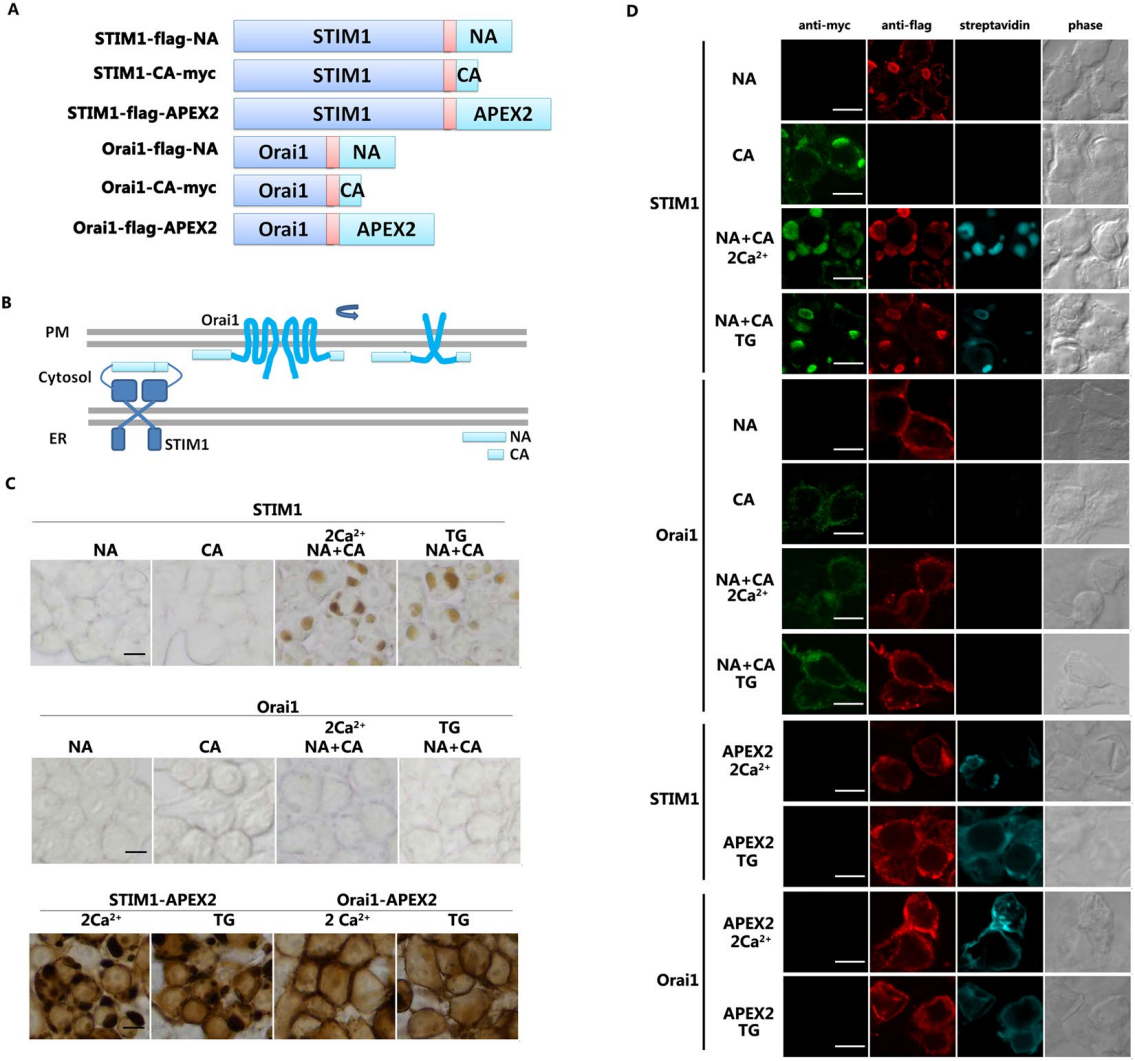
Examination of homo-dimerization of STIM1 using protein-fragment complementation (PFC) of APEX2. (**A**) Schematic of STIM1-NA, STIM1-CA, STIM1-APEX2, ORAI1-NA, ORAI1-CA and ORAI1-APEX2. APEX2 and NA are tagged with flag, while CA is tagged with myc. (**B**) Schematic of the topology of STIM1-PFC and ORAI1-PFC of APEX2. (**C**) HEK-293 cells were transfected with various plasmids as indicated. NA indicates STIM1 or Orai1 fused with flag-NA, CA indicates STIM1 or Orai1 fused with CA-myc. Cells were treated with TG or 2Ca^2+^ for 15 minutes. DAB staining was performed as in Fig. 1(D). Scale bar: 10 μm. (**D**) HEK-293 cells plated on glass were transfected with different plasmids as indicated. Live cells were stimulated with TG or DMSO as above. Biotin labeling was initiated as in Fig. 1(E). Scale bar: 10 μm.

STIM1 is a key modulator of the store operated calcium channel entry (SOCE) at ER. It acts as an ER-resident Ca^2+^ sensor that can be activated by the depletion of ER Ca^2+^ stores^24^. STIM1 was reported as a dimer in the resting and activated states^25^. Because the C-terminus is composed of flexible fragments, we tested whether this could help the reassembly of the enzyme. We fused NA or CA to the cytoplasmic C-terminus of STIM1 to construct STIM1-NA and STIM1-CA (Fig. 2A,B). When we co-transfected the constructs into HEK-293 cells, there was a significant DAB signal both in the resting condition and after depletion of Ca^2+^ stores with thapsigargin (TG), while no signal was detectable over the background with single mutant expression (Fig. 2C upper). For comparison, we transfected the cells with STIM1-APEX2 and detected a strong signal condensed below the plasma membrane and some mild signals in the cytoplasm (Fig. 2C bottom, left). However, the signal produced by the STIM1-NA/STIM1-CA was more specific.

**Figure 3:**
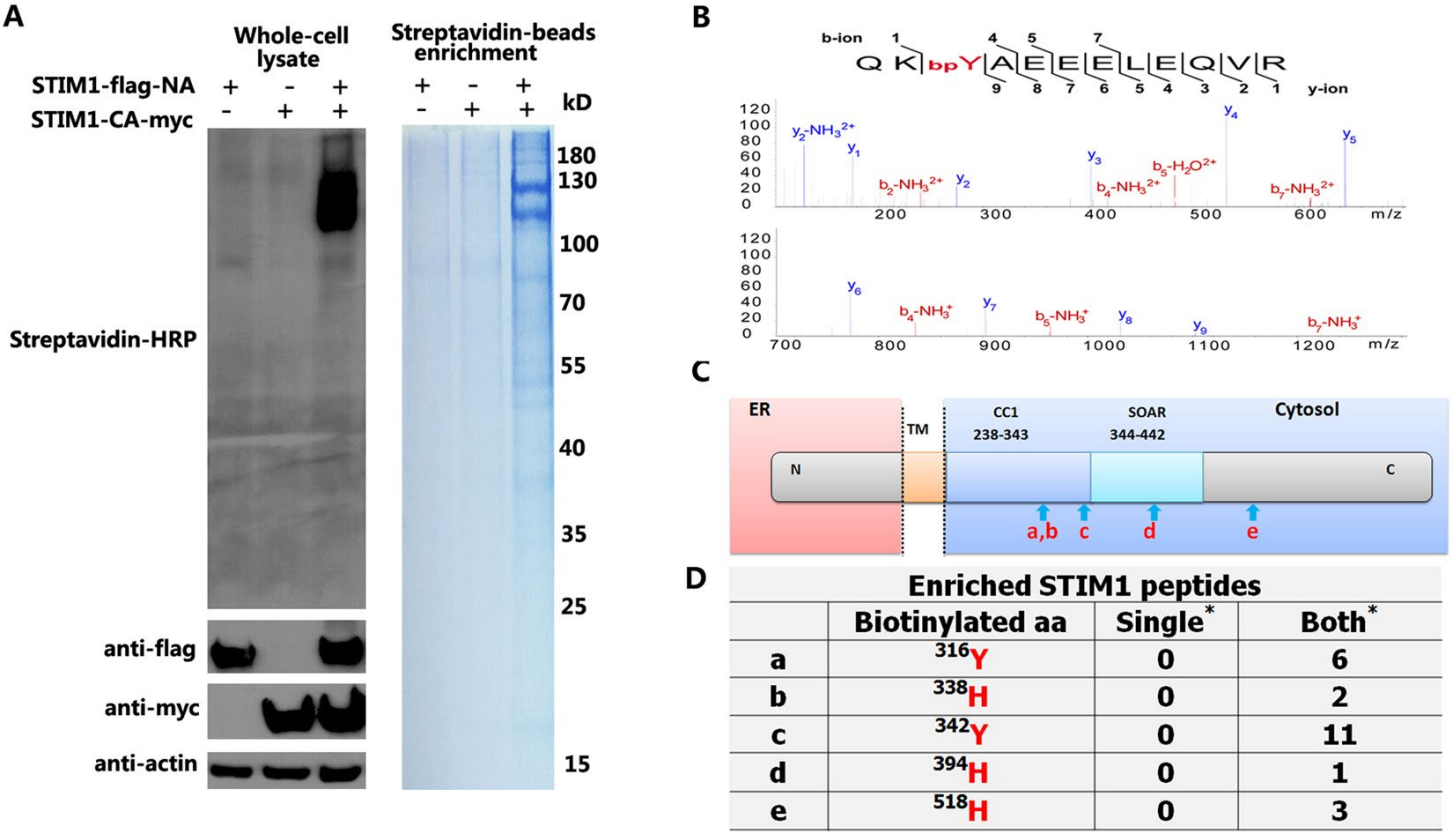
Biotinylation analysis of reconstituted enzyme by STIM1-NA/CA. (**A**) The cells were transfected with the different plasmids as indicated. Biotinylation was initiated as in Fig. 1(E). Whole cell lysates were resolved by 10% SDS-PAGE, and detected by western blot using streptavidin-HRP, anti-flag, anti-myc and anti-actin (left). The original blots were presented in the Supplementary Fig. S4. Enrichment was performed with streptavidin-beads, and proteins were separated by SDS-PAGE gel (right). These experiments were separately repeated twice. (**B**) Example of an MS/MS spectrum for a peptide with biotin-phenol on tyrosine. Fragment ions assigned to both y-ions and b-ions are labeled. (**C**) Schematic of the topology of different domains in STIM1. Red letters a-e indicate the approximate positions of identified biotin-labeled sites. (**D**) The biotin-labeled sites and their counts of the MS2 spectra. Each existing biotinylated amino acid is shown in red. Single*: cells expressed only STIM1-NA or STIM1-CA; Both*: cells expressed both STIM1-NA/STIM1-CA, which were in 2 mM calcium.

To evaluate the biotin labeling efficiency, we incubated the cells expressing the PFC of APEX2 with biotin-phenol (BP) and initiated with H_2_O_2_. A similar distribution of the biotinylated proteins was produced to that of DAB in cells co-expressing both STIM1-NA and STIM1-CA with or without TG; there was no detectable signal in the cells expressing either mutant alone. The localized staining pattern was apparent compared to the widely dispersed signal produced by cells expressing STIM1-APEX2 (Fig. 2D). An independent western blot experiment further confirmed the biotinylation capability reconstituted by STIM1-NA and STIM1-CA (Fig. 3A). These results further demonstrate that the complementary fragments can reconstitute the APEX2 enzyme when they are brought together in close proximity.

### Orai1 homodimer

Orai1, located on the plasma membrane, is another crucial pore-forming component of the SOCE channel. Orai1 has been reported as a dimer in the resting state and a tetramer or hexamer when activated^26–28^. The structure-function study shows that each Orai1 has 4 transmembrane helices (TM1-4) which are linked with the N and C extensions that face the cytosol. TM1 lines the pore, while TM4 and the downstream C-terminus are located at the periphery of the gate with an anti-parallel orientation between two isoforms^28^. We fused the NA and CA to the C-terminus of Orai1 to check whether or not the far distance of the Orai1 C- terminus would prevent reconstitution of the enzyme activity (Fig. 2A,B). Both the DAB staining and biotin labeling experiment showed that no signal was detectable when Orai1-NA and Orai1-CA were co-expressed with or without TG stimulation, while cells expressing Orai1-APEX2 exhibited a strong signal mainly at the plasma membrane (Fig. 2C,D). These results demonstrate that the complementary pair can not reconstitute the enzyme when fused to the Orai1 C-terminus. The failure to reconstitute the enzyme may result from the large distance between the APEX2 pairs fused to Orai1; it is suggested from the crystal structure of Orai1 that the C-termini of the neighboring Orai1s form an alternating pair^28,29^. These results provide further evidence that the reconstitution by the PFC of APEX2 requires a close distance and orientation match in three-dimensional space.

### Biotin enrichment and identification of the binding sites in the STIM1 homodimer

To further exploit the advantages of APEX2-based proximity-labeling, we aimed to identify the nearby proteins of the STIM1 dimer through biotin labeling, affinity purification using streptavidin beads and subsequent MS analysis. The biotinylation efficiency was verified by western blot with HRP-streptavidin and further confirmed by affinity enrichment using streptavidin beads (Fig. 3A). The gels were subjected to in-gel digestion with trypsin, and the resulting peptides were analyzed by MS. All results for the biotinylated peptides were confirmed by manually checking the MS/MS spectra. In total, 36 biotinylated peptides were specifically identified in STIM1-NA/STIM1-CA co-expressing cells, rather than in either the STIM1-NA or STIM1-CA expressing cells, of which there were 23 peptides belonged to STIM1 itself, representing five different biotinylated sites (Supplementary Table S1). This indicated that the high specificity of biotinylation catalyzed by the reassembled peroxidase. Moreover, further mapping of these five biotinylated sites revealed that all of them were localized to the cytoplasmic domains of STIM1 (Fig. 3C). While one site was located in the C-terminal flexible region (^518^hSDSESSLHmSDR), the other four sites, located in the 316-to-394 amino acid region, were sterically adjacent to each other according to the reported STIM1 fragment structure of CC1-CC2 and SOAR^30^. These results strongly demonstrate that the complementary STIM1-NA and STIM1-CA can fully reconstitute the peroxidase activity and biotinylate proteins that are nearby.

## Discussion

Protein-protein interactions play vital roles in the life of the cell. The PFC assay is a useful strategy that can provide high specificity and sensitivity because of its absolute requirement for the correct three-dimensional structure of a resultant fragment hybrid^11^. In this study, we developed a PFC of APEX2 based on a readily observable biological readout, DAB staining. To achieve this, we fused N-terminal APEX2 (NA) to FRB and C-terminal APEX2 (CA) to FKBP12. The NA and CA do not spontaneously reassemble. The interaction between FRB and FKBP12 induced by rapamycin can force the reconstitution of the two halves of APEX2, leading to full enzyme activity that can biotinylate neighboring proteins and catalyze the DAB polymer(Fig. 1, Supplementary Fig. S2). We identified 3 NAs (NA201, NA215, NA230) that could complement to CA202.

The 201^th^ amino acid lies in the 14^th^ turn between the 11^th^ and 12^th^ alpha helix, the 215^th^ amino acid lies in the 15^th^ turn, and the 230^th^ amino acid lies at the beginning of the 14^th^ alpha helix. Other N-terminal mutants that were larger than these three could not show the enzyme activity (Supplementary Fig. S1) when coupled with CA202. These results demonstrate that the correct secondary structure of the N-terminus is essential for its function. It is thus essential to choose the proper truncation site that will not disrupt the 3D structure of the PFC pair.

SOCE mediated by STIM1 and ORAI1 plays a critical role in calcium homeostasis in most mammalian cells. Both STIM1 and ORAI1 can form homodimers in the resting and active states. We fused the complementary APEX2 pair to each of their C-termini and obtained a space-limited signal in the STIM1 homodimer but not in the ORAI1 homodimer, suggesting the specificity of the PFC of APEX2. Many studies using the STIM1 cytosolic domain have made clear that the CCI and SOAR regions are responsible for the intermolecular interaction and that the C-terminal region remains flexible^31,32^. These features are consistent with the successful reconstitution of enzyme activity by attaching the PFC pair of APEX2 to the C-terminus of STIM1 and with biotinylation near the interaction region. Although the Orai1 homodimer may bring the complementary fragments close in the resting and activate states, the C-terminus of Orai1 that forms alternating pairs may enlarge the distance of the complementary pairs, thereby leading to the failed reassembly of the enzyme activity.

The limitation of PFC of APEX2 is that the reconstituted enzyme activity may be relatively lower than APEX2. We did not identify STIMATE, a protein reported to physically bind to the STIM1 juxtamembrane CC1 instead of the SOAR domain. This is probably because the distance between STIMATE and STIM1 is beyond the active region of the reconstituted enzyme^33^. In addition, the localization of the complemented pair, expression level and the expression ratio should be considered when assessing the results.

In conclusion, the PFC of APEX2 that we identified can reconstitute full enzymatic activity when the domains are directed in proximity to each other. This strategy enhances the specificity of the proximity-tagging method where biotinylation only occurs at the right time and the right site when the protein complex is formed. By taking advantage of the unstable nature of the phenoxyl radical generated during oxidation of the reconstituted enzyme, we can obtain the limited interaction regions between proteins under a specific condition in live cells.

## Methods

### Chemicals and reagents

PCDNA3-flag-APEX2 was purchased from Addgene (Cambridge). The following reagents were purchased from Sigma: rapamycin, DAB, 30% (w/w) hydrogen peroxide (H_2_O_2_), the anti-flag antibody, and standard reagents for gel electrophoresis and western blotting including Ponceau S HCl, urea, biotin, the 100x protease inhibitor cocktail, sodium deoxycholate, sodium dodecyl sulfate, sodium ascorbate, Trolox, and sodium azide. The anti-myc antibody was purchased from Cell Signaling Technology. Streptavidin-HRP was purchased from CWBIO. Biotin-phenol was purchased from Iris-Biotech (CAS-NO: 41994-02-9). Lipofectamine 2000 was purchased from Life Technologies. Coomassie and sequencing grade modified trypsin were purchased from Promega. Streptavidin magnetic beads were purchased from Pierce (Catalog No. 88817).

### Construction of incremental truncations and interaction protein pairs

The initial N- and C-terminal fragments of APEX2 were amplified from PCDNA3-flag-APEX2. The rapamycin-binding domain (FRB) of human mTOR and human FK506-binding protein 12 (FKBP) were generated by PCR amplification from plasmids in our lab. A flexible linker was added as described^12^. Briefly, to separate the mutant and targets, we introduced linker 1 (QISYASRGGGSSGGG) for NA and linker 2: (GGGSSGGGQISYASRG) for CA. All mutants and the wild-type flag-APEX2 were amplified by PCR to tag targets as indicated.

### Cell culture, transfection and treatment

HEK-293 cells were cultured in DMEM supplemented with 10% FBS at 37 °C with 5% CO2. Transfections were performed as described in the protocol. Briefly, cells were seeded to different plates, and the next day, the DNA and Lipofectamine were mixed and added to the cells. Forty hours later, expression was detected by western blot or immunofluorescence. For imaging experiments, cells were grown on small glass coverslips that were pretreated with 10 μg/ml polylysine for 20 minutes in 24-well plates. For the STIM1 activation, cells transfected with indicated plasmids were treated with either 2-Ca^2+^ Ringer’s (155 mM NaCl, 4.5 mM KCl, 2 mM CaCl2, 1 mM MgCl2, 10 mM D-glucose, and 5 mM Hepes, pH 7.3 with NaOH) or 0-Ca^2+^ Ringer’s (155 mM NaCl, 4.5 mM KCl, 3 mM MgCl2, 10 mM D-glucose, 5 mM Hepes, and 1 mM EGTA, pH 7.3.) plus 1 μM TG for 15 minutes^24^. To stimulate the interaction between FRB and FKBP12, 200 nM rapamycin were added 6 hours before experiments.

### DAB staining

DAB staining was performed on transiently transfected HEK-293 cells as described^34^. Briefly, 40 hours after the transfection, cells were washed with PBS and fixed with 2% glutaraldehyde (Electron Microscopy Sciences) in PBS buffer, pH 7.4 for 30 minutes. Then, cells were rinsed with PBS twice and quenched with 20 mM glycine in PBS for 5 minutes, followed by 2 washes with PBS. DAB staining was initiated with the addition of freshly diluted 0.5 mg/ml DAB with 10 mM H_2_O_2_ in PBS. After 15–30 minutes, reactions were stopped by removal of DAB with two washes. Cells were imaged by bright-field microscopy. Acquisition times ranged from 50–100 ms.

### Image analyses

All acquired images were analyzed using the ImageJ (NIH) software.

### Biotin-phenol labeling in live cells

Biotin-phenol (BP) labeling was performed as described^9^. Briefly, genes were transfected into HEK-293 cells using Lipofectamine 2000. After 40 hours, biotin-phenol labeling was initiated by the addition of 0.5 mM BP, which was incubated at 37 °C in 5% CO2 for 30 minutes, accompanied by the addition of 1 mM H_2_O_2_ for 1 minute. The reaction was stopped with quenching buffer containing 10 mM sodium azide, 10 mM sodium ascorbate, and 5 mM Trolox. Cells were washed 6 times with PBS containing the 3 inhibitors followed by 3 washes with PBS. All washes were performed on ice. Cells were then detected by immunofluorescence or western blot.

### Immunofluorescence microscopy

After initiations with BP and H_2_O_2_, cells were washed with ice-cold quenching buffer and fixed with 4% PFA for 20 min at room temperature (RT) followed by permeabilization with PBS with 0.1% Triton(PBST) for 5 minutes. They were then blocked with 3% BSA in PBS. The NA was detected with the flag antibody, CA was detected with anti-myc, and the biotin signal was detected with cy5-streptavidin after one hour incubations at RT or overnight incubations at 4 °C. After three washes with PBST, cells were incubated with secondary Alexa Fluor 488-goat anti-mouse IgG or Alexa Fluor cy3-goat anti-rabbit IgG for 1 h at RT. Cells were washed six times with PBST. Confocal imaging was performed using an Olympus FV1200. The confocal head contained a quad-band notch dichroic mirror (405/488/561/647 nm).

### Western blot analysis

Biotinylated cells were washed, and whole-cell lysates were prepared in RIPA buffer (50 mM Tris, 150 mM NaCl, 0.1% SDS, 0.5% sodium deoxycholate, 1% Triton X-100, 1 × protease cocktail, 1 mM PMSF, 10 mM sodium azide, 10 mM sodium ascorbate, and 5 mM Trolox). After centrifugation to remove insoluble materials, proteins were separated by SDS-PAGE and analyzed using streptavidin-HRP, anti-flag antibody, and anti-myc antibody. A small fraction of the cells that were washed and lysed without the quenchers was used for protein concentration measurements by BCA.

After washing, cells were washed with the quencher solution for six times, lysed with 800 μL of freshly prepared RIPA lysis buffer on ice, and ultra-sonicated. Lysates were clarified by centrifugation at 13,000 rpm for 10 minutes at 4 °C. Then, we put the supernatants into 3k columns (balanced with RIPA first), centrifuged at 13,000 rpm for 30 minutes at 4 °C and washed the columns with RIPA 6 times. The sample was thoroughly recovered to a new EP tube. The suspensions were incubated with 150 μL streptavidin-coated magnetic beads at room temperature for 1 hour. Streptavidin beads were then washed with 2 × 1 mL RIPA lysis buffer, once with 1 M KCl, 0.1 M Na_2_CO_3_ and 2 M Urea, and again with 2 × 1 mL RIPA lysis buffer. Biotinylated proteins were eluted by incubating the beads with 40 μL 1x NuPAGE LDS Sample Buffer supplemented with 20 mM DTT and 2 mM biotin and heating to 95 °C for 5 minutes.

### In-gel protein digestion and extraction

Enriched biotinylated proteins by magnetic streptavidin beads were separated on 10% SDS-PAGE gels and stained with Coomassie G-250 (Invitrogen). Lanes for each replicate were manually cut into 3–4 gel bands. Each band was destained with 40% acetonitrile/50 mM NH_4_HCO_3_. The gels were dehydrated with 100% acetonitrile and dried for 5 min using a SpeedVac. Disulfide bonds were reduced with DTT (10 mM, 56 °C, 45 min), and the free sulfhydryl groups were alkylated with iodoacetamide (55 mM, 25 °C, 60 min in the dark). Gel pieces were sequentially washed with 50 mM NH_4_HCO_3_, 50% acetonitrile/50 mM NH_4_HCO_3_, and dehydrated with 100% acetonitrile. After drying with a SpeedVac, the gel was rehydrated using 100 ng/μL trypsin (Promega, V5113) on ice for 30 min, and the digestion was carried out at 37 °C overnight and quenched with 1.0% TFA. The tryptic peptides were extracted twice with 60% acetonitrile containing 0.1% TFA, and the combined digest solution was dried using a SpeedVac.

### Mass spectrometry

The nano-LC-MS/MS experiments were performed on a Q Exactive mass spectrometer (Thermo Scientific) coupled to an Easy-nLC 1000 HPLC system (Thermo Scientific). The dried peptides were resuspended in 0.1% formic acid/2% acetonitrile and loaded onto a 100 μm id × 2 cm-fused silica trap column packed in-house with reversed-phase silica (Reprosil-Pur C18 AQ, 5 μm, Dr. Maisch GmbH) and then separated on a 75 μm id × 20 cm C18 column packed with reversed-phase silica (Reprosil-Pur C18 AQ, 3 μm, Dr. Maisch GmbH). The loaded peptides were eluted with a 78-min gradient. Solvent A consisted of 0.1% FA in a water solution, and solvent B consisted of 0.1% FA in an acetonitrile solution. The segmented gradient was 4–12% B, 5 min; 12–22% B, 50 min; 22–32% B, 12 min; 32–95% B, 1 min; 95% B, 7 min at a flow rate of 280 nl/min.

The mass spectrometer was operated in the data-dependent acquisition mode, and full-scan MS data were acquired in the Orbitrap with a resolution of 70,000 (m/z 200) across the mass range of 300–1600 m/z. The target value was 3.00E + 06 with a maximum injection time of 60 ms. The raw MS data were processed with Proteome Discovery (version 1.4, Thermo Scientific). Briefly, peptide identification was performed with the Sequest HT search engine against a UniProt database supplemented with all the frequently observed contaminants in MS. The following parameters were used for database searching: 10 ppm precursor mass tolerance, 0.02 Da fragment ion tolerance, up to two missed cleavages, carbamidomethyl cysteine, biotin-phenol on tyrosine, histone, cysteine and tryptophan, and oxidized methionine as the variable modification. The peptide confidence was set at a high level (q-value < 0.01) for the peptide filter. A manual check was performed for the biotinylated peptides to avoid suspicious assignments with unclear MS/MS spectra.

## Electronic supplementary material

Supplementary Figure

Supplementary Table S1

Supplementary raw blot data
