# Author Correction: Optimizing the fragment complementation of APEX2 for detection of specific protein-protein interactions in live cells

**DOI:** 10.1038/s41598-018-24790-5

**Published:** 2018-04-20

**Authors:** Miaomiao Xue, Junjie Hou, Linlin Wang, Dongwan Cheng, Jingze Lu, Li Zheng, Tao Xu

**Affiliations:** 1 National Laboratory of Biomacromolecules, CAS Center for Excellence in Biomacromolecules, Institute of Biophysics, Chinese Academy of Sciences, Beijing, 100101 China; 2 College of Life Sciences, University of Chinese Academy of Sciences, Beijing, 100049 China

Correction to: *Scientific Reports* 10.1038/s41598-017-12365-9, published online 27 September 2017

In the original version of this Article, Affiliations 1 and 2 were not listed in the correct order. The correct affiliations are listed below:

Affiliation 1

National Laboratory of Biomacromolecules, CAS Center for Excellence in Biomacromolecules, Institute of Biophysics, Chinese Academy of Sciences, Beijing, 100101, China.

Affiliation 2

College of Life Sciences, University of Chinese Academy of Sciences, Beijing, 100049, China.

This error has now been corrected in the PDF and HTML versions of the Article and in the supplementary information.

