# Supplementary material for: Optimizing the fragment complementation of APEX2 for detection of specific protein-protein interactions in live cells
Source: Sci Rep. 2017 Sep 27;7:12039. doi: 10.1038/s41598-017-12365-9 (PMC5617831; doi:10.1038/s41598-017-12365-9)
Supplement: Supplementary file 1 — Supplementary Figure [file 41598_2017_12365_MOESM1_ESM.pdf]

Optimizing the fragment complementation of APEX2 for detection of specific protein-protein interactions in live cells.

Miaomiao Xue[1, 2*], Junjie Hou[1,*], Linlin Wang[1,2], Dongwan Cheng[1], Jingze Lu[1], Li Zheng[1,+], Tao Xu[1,2+]

1. National Laboratory of Biomacromolecules, CAS Center for Excellence in Biomacromolecules, Institute of Biophysics, Chinese Academy of Sciences, Beijing 100101, China
2. College of Life Sciences, University of Chinese Academy of Sciences, Beijing 100049, China

+ Correspondence: Li Zheng, E-mail: zhengli@ibp.ac.cn, Tao Xu, E-mail: xutao@ibp.ac.cn

* The two authors contributed equally to the article.

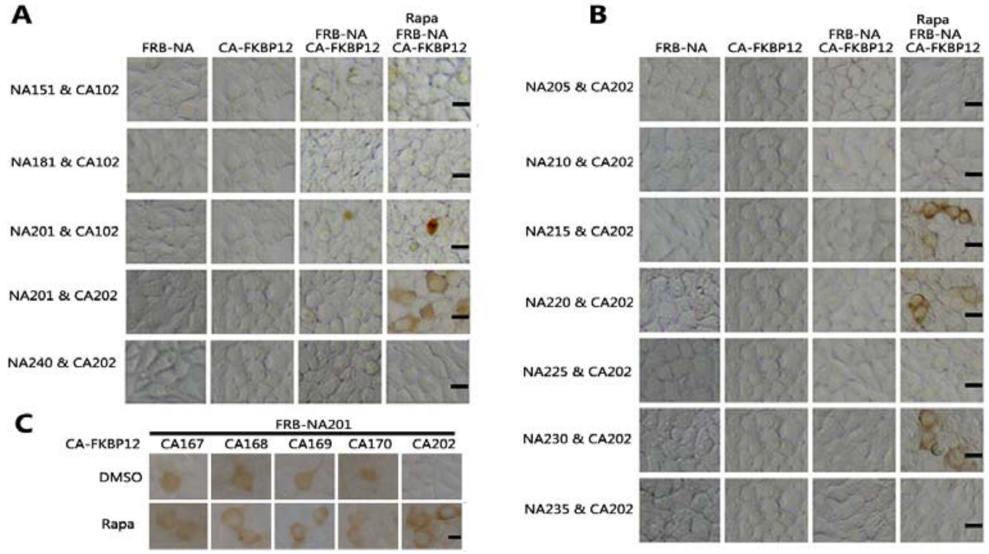

Supplementary Figure. S1. Screening for the optimal complementary APEX2 mutant pairs. (A) First stage of screening. We started from NA151, NA181, NA201 and NA240 as FRB-NA, together with CA102 and CA202 as CA-FKBP12. Cells were transfected with single or both mutants as shown. The different pairs of NA and CA are shown on the left. Forty hours later, cells were treated with DMSO or rapamycin followed by DAB staining. (B) and (C), Second stage of screening. (B) Different FRB-NA combined with C202-FKBP12. Cells were transfected with single or both mutants as shown. Forty hours later, cells were treated with DMSO or rapamycin followed by DAB staining. (C) Different CA-FKBP12 combined with FRB-NA201. Cells were co-transfected with different combinations of mutants treated with DMSO or rapamycin as indicated. DAB staining was performed as in Fig. 1. Scale bar: 10 µm.

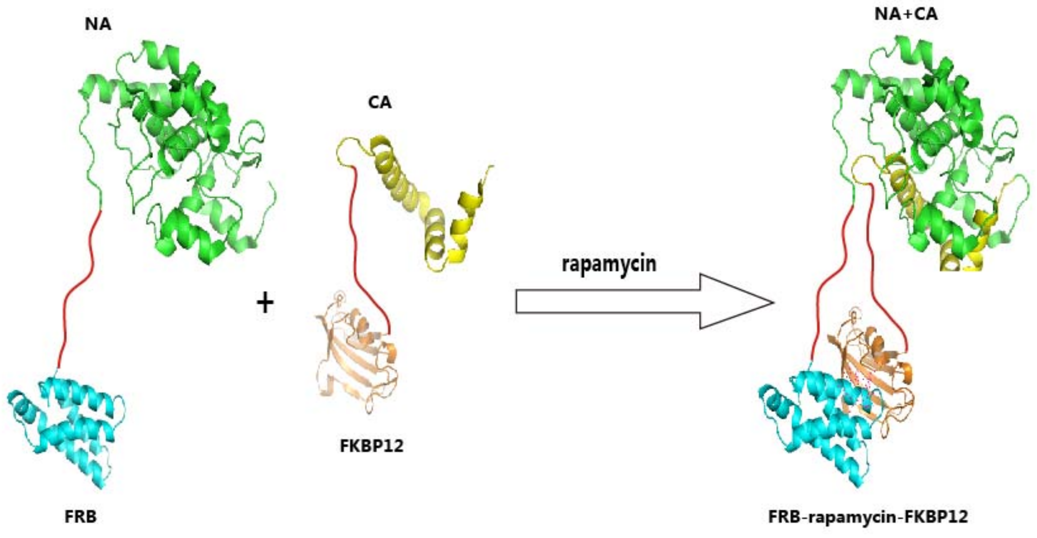

Supplementary Figure. S2. A schematic diagram from two publically available co-crystal structures, APEX (1OAG)[1] and mTOR FRB-domain–rapamycin–FKBP12 (1FAP)[2]. NA(green) links with FRB(blue) by linker1(red) and CA(yellow) links with FKBP12(orange) by linker2(red). Rapamycin induces a tight interaction between FRB and FKBP12 which leads to the reconstitution of APEX2 by NA and CA.

1 Sharp, K. H., Mewies, M., Moody, P. C. & Raven, E. L. Crystal structure of the ascorbate peroxidase-ascorbate complex. Nat Struct Biol. **10**, 303-307 (2003).

2 Choi, J., Chen, J., Schreiber, S. L. & Clardy, J. Structure of the FKBP12-rapamycin complex interacting with the binding domain of human FRAP. Science. **273**, 239-242 (1996).
