# Supplementary material for: Optimizing the fragment complementation of APEX2 for detection of specific protein-protein interactions in live cells
Source: Sci Rep. 2017 Sep 27;7:12039. doi: 10.1038/s41598-017-12365-9 (PMC5617831; doi:10.1038/s41598-017-12365-9)
Supplement: Supplementary file 3 — Supplementary raw blot data [file 41598_2017_12365_MOESM3_ESM.pdf]

Optimizing the fragment complementation of APEX2 for detection of specific protein-protein interactions in live cells.

Miaomiao Xue[1, 2*], Junjie Hou[1,*], Linlin Wang[1,2], Dongwan Cheng[1], Jingze Lu[1], Li Zheng[1,+], Tao Xu[1,2+]

1. National Laboratory of Biomacromolecules, CAS Center for Excellence in Biomacromolecules, Institute of Biophysics, Chinese Academy of Sciences, Beijing 100101, China
2. College of Life Sciences, University of Chinese Academy of Sciences, Beijing 100049, China

+ Correspondence: Li Zheng, E-mail: zhengli@ibp.ac.cn, Tao Xu, E-mail: xutao@ibp.ac.cn

* The two authors contributed equally to the article.

The original blots for the cropped blots in the body of paper.

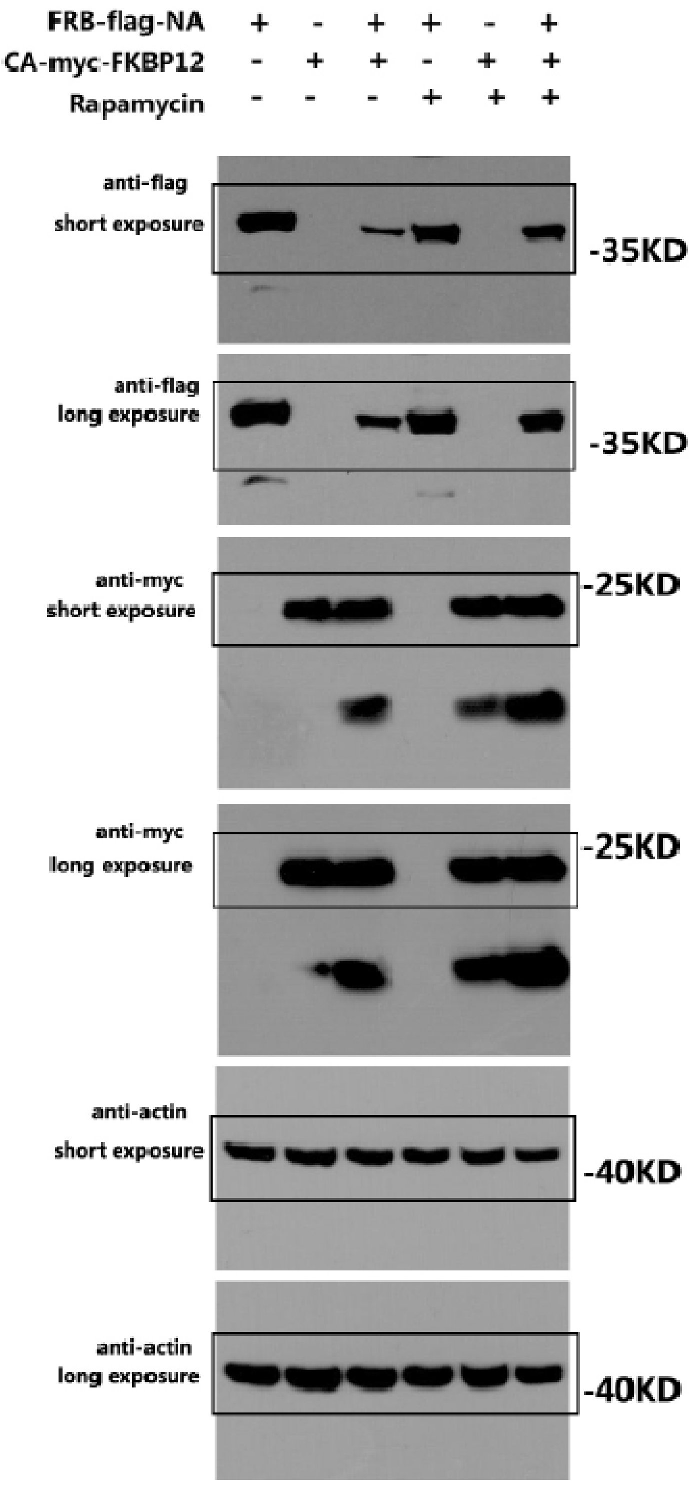

Supplementary Figure S3. Cells were co-transfected with different combinations of plasmids and treated with DMSO or rapamycin. The whole cell lysates were resolved by 12% SDS-PAGE gel and transferred to a membrane. We cut the membrane according to the expected molecular length of different interested proteins to perform immunodetection with respective antibodies. The expression of FRB-flag-NA were detected by anti-flag antibody, CA-myc-FKBP12 were detected by anti-myc antibody and actin were used as a protein loading control for each lane. The coding sequence of FRB-flag-NA is 1008 bp and CA-myc-FKBP12 is 615 bp.

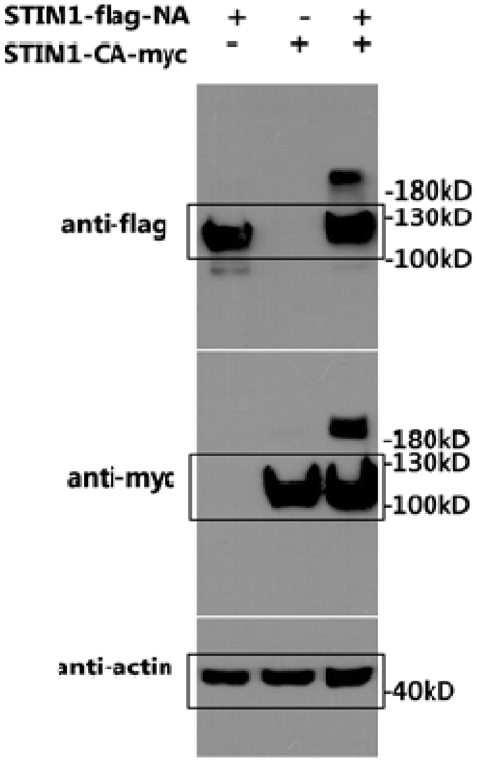

Supplementary Figure S4. Cells were co-transfected with different combinations of plasmids. 40 hours later, the whole cell lysates were resolved by 10% SDS-PAGE gel and transferred to a membrane. We cut the membrane according to the expected molecular length of different interested proteins to perform immunodetection with respective antibodies. The expression of STIM1-flag-NA were detected by anti-flag antibody, STIM1-CA-myc were detected by anti-myc antibody and actin were used as a protein loading control. The coding sequence of STIM1-flag-NA is 2757 bp and STIM1-CA-myc is 2346 bp.
